# A Single-Center Case Series of Acute Retinal Necrosis at Teikyo University: Clinical Characteristics and Treatment Outcomes

**DOI:** 10.7759/cureus.62343

**Published:** 2024-06-13

**Authors:** Tatsuya Mimura, Atsushi Mizota, Emiko Watanabe, Gaku Terauchi, Makoto Kawashima, Yuji Inoue

**Affiliations:** 1 Department of Ophthalmology, Teikyo University School of Medicine, Tokyo, JPN; 2 Department of Ophthalmology, Nishikasai Inouye Eye Hospital, Tokyo, JPN

**Keywords:** vitrectomy, varicella-zoster virus, polymerase chain reaction, herpes simplex virus, acute retinal necrosis

## Abstract

Aim

To evaluate the clinical characteristics, treatment course, and prognosis of patients with acute retinal necrosis (ARN), which can rapidly progress and cause severe vision loss.

Design

Single-center retrospective case series.

Subjects and methods

Six patients and seven eyes diagnosed with ARN at Teikyo University Hospital were included in this study. The clinical presentation and treatment prognosis were investigated based on data obtained from medical records.

Results

The mean age of the patients at the initial diagnosis was 63.6 years. Although the mean Logarithm of the Minimum Angle of Resolution (LogMAR) visual acuity tended to decrease from 0.77 at the first visit to 1.29 at the last visit, the difference was not statistically significant. Intraocular manifestations observed during the study period included ocular hypertension (14.3%), anterior uveitis (100.0%), retinal hemorrhage (71.4%), vitreous opacity (100.0%), retinal exudative vasculitis (85.7%), optic nerve atrophy (85.7%), retinal vascular occlusion (85.7%), choroidal atrophy (85.7%), macular edema (100.0%), subretinal fluid in the macula (71.4%), and retinal detachment (85.7%). Treatment modalities included oral and intravitreal antivirals (85.7%), antiplatelet medications (85.7%), steroid eye drops (85.7%), subcapsular (57.1%) and vitreous (42.9%) steroid injections, oral steroids (71.4%), and surgical intervention (85.7%). Vitrectomy led to retinal recovery in all five eyes that underwent the procedure.

Conclusions

The visual prognosis of patients with ARN is poor, particularly in those with preexisting visual impairment. Early detection coupled with antiviral therapy and prompt surgical intervention have been identified as potential factors that influence visual outcomes. Given the severity of ARN, collecting data from multiple centers could aid in devising future diagnostic and therapeutic strategies.

## Introduction

Acute retinal necrosis (ARN) is a retinal disorder characterized by vascular impairment of the retina resulting from intraocular infection by varicella-zoster virus (VZV), herpes simplex virus (HSV) 1/2, or cytomegalovirus (CMV) [1,2]. The clinical features of ARN include acute visual disturbances, ocular pain, retinal inflammation, and loss of the visual field due to progressive retinal necrosis. This disease is prevalent among immunocompromised individuals, such as those on immunosuppressive drugs or those infected with the human immunodeficiency virus (HIV) [3]. It is also associated with certain viral infections, primarily herpes viruses, including HSV [4].

ARN presents a significant ophthalmic challenge owing to its relentless progression and rapid decline in visual function. Therefore, research into the clinical manifestations and treatment outcomes of ARN is of vital interest to ophthalmologists. Moreover, a comprehensive understanding of the clinical features exhibited by affected individuals and the efficacy of treatment modalities has direct implications for the visual prognosis and overall management strategies adopted for patients with ARN.

Numerous case reports regarding ARN have emerged from diverse medical institutions across Japan [5,6,7,8]. However, large, comprehensive investigations of ARN remain scarce. Consequently, the establishment of standardized treatment protocols continues to elude the field [9,10]. ARN, a severe ailment necessitating immediate intervention, is often initiated based on clinical symptoms. Our main purpose was to evaluate the clinical characteristics, treatment course, and prognosis of patients with ARN. The aggregation of data from multiple healthcare facilities holds promise for shaping future diagnostic and therapeutic strategies, thereby fostering the formulation of robust global guidelines. Therefore, the present study aimed to delineate the clinical attributes of ARN cases within the purview of Teikyo University while assessing their congruence with the existing literature.

## Materials and methods

This study was conducted at Teikyo University Hospital, Tokyo, Japan, in accordance with the ethical guidelines of the Declaration of Helsinki [11] and the Ethical Guidelines for Medical Health. This study was retrospectively approved by the Teikyo University Hospital Research Ethics Committee (Teirin 18-061), and the requirement for written informed consent was waived. We conducted a comprehensive review of all patients diagnosed with and managed for ARN at our institution from January 1, 2010, to December 31, 2023.

The eligibility criteria stipulated the inclusion of patients aged 18 years and older with confirmed ARN while excluding those with concurrent ocular fundus disorders or traumatic ocular injuries. Consequently, adherence to the predetermined inclusion criteria yielded a cohort of seven eligible patients with no instances of exclusion. Subsequently, the complete enumeration included a final tally of seven patients. Meticulous curation of the patients' medical records was achieved through a rigorous retrospective collection and thorough review process.

The diagnosis of ARN was meticulously made through clinical assessment, rigorously adhering to the guidelines outlined by the American Uveitis Study Group [12].

The grading of ARN severity adhered to the classification system delineated in the seminal work by Tokyo Medical University [13], a pivotal reference point frequently employed in Japanese ARN research. The severity stratification was as follows: Grade 1: mild type, defined by the presence of localized lesions exhibiting a gradual progression pattern; Grade 2: severe type, manifests as circumscribed lesions with a progression rate notably slower than that observed in fulminant cases; Grade 3: fulminant type, characterized by the presence of yellowish-white retinal lesions extending to the posterior pole within a 10-day window after symptom onset.

The present study analyzed various clinical parameters, including demographic factors, such as sex and age at onset; ocular manifestations, including the affected eye; and initial examination findings, such as visual acuity. Additionally, a detailed examination was conducted on distinctive clinical features such as anterior uveitis with granulomatous membrane, fundus hemorrhage, vitreous opacity, the presence of exudative vasculopathy, retinal detachment (RD), optic nerve atrophy, retinal vascular occlusion, and retinal degeneration and atrophy. Furthermore, the time from the onset of ARN to the start of treatment, surgical procedures, the presence of retinal reattachment, and final visual acuity were examined.

## Results

Between 2010 and 2023, six cases of ARN were diagnosed in seven eyes. Six of these eyes underwent surgery, and one eye was treated with medical therapy.

Patient characteristics are shown in Table 1. The mean age was 44.3 ± 12.9 years. There were two males and four females. Systemic diseases included hypertension in three patients, hyperlipidemia in one patient, and organic brain disease (Alzheimer's) in one patient.

**Table 1:** Patient characteristics

Eye No.	Age	Male/Female	Eye (Right/Left)	Hypertension	Diabetes	Hyperlipidemia	Heart disease	Brain disease	Others
1 2	59	F	R/L	0	0	0	0	0	Arthritis, thrombotic varicose veins of lower extremities
3	43	M	R	0	0	0	0	0	0
4	67	M	R	1	0	0	0	0	0
5	70	F	R	0	0	0	0	0	0
6	70	F	R	1	0	0	0	0	0
7	73	F	R	1	0	1	0	1	Alzheimer's disease

The results of the viral antibody (Ab) titers and serological tests at baseline are shown in Table 2. HSV1 was measured in all patients, while HSV2 was measured in only one patient. The reason for this is that HSV2 measurement is not part of the hospital's routine testing. All cases were positive for HSV and VZV, with antibody titers of 4 or higher. One patient tested positive for the syphilis surface antigen Treponema pallidum (TP)-Ab and HIV.

**Table 2:** Viral antibody titers and serologic tests at baseline HSV1/2: herpes simplex virus type 1/2; VZV: varicella-zoster virus; CMV: cytomegalovirus; PHA: phytohemagglutinin; HIV: human immunodeficiency virus; ANA: anti-nuclear antibody; STS: serologic test for syphilis; TP: Treponema pallidum; HBS Ag: hepatitis B surface antigen; s/co: (value of specimen/cut-off value); HCV Ab: hepatitis C virus antibody; CH50: 50% hemolytic complement activity; IL2-R: interleukin-2 receptor; CLIA: chemiluminescent immunoassay; -: not tested

	HSV1 (U)	HSV2 (U)	VZV (U)	CMV (U)	Toxoplasma IgG (U)	Toxoplasma PHA (U)	HIV (copy/ml)	ANA (U)	STS (U)	TP Ab (U)	HBS Ag (s/co)	HCV Ab (s/co)	CH50 (U/mL)	IL-2R (U/ml)
Normal range	<4	<4	<4	<4	<20	<160	0	<40	0	0	<0.05 (CLIA)	<10.0 (CLIA)	30～45	145～ 519
Eye No.														
1	4	-	4	16.6	7.5	-	0	-	0	0	0.1	0.1	60	-
2	4	-	4	16.6	7.5	-	0	-	0	0	0.1	0.1	60	-
3	4	4	16	4	5	160	1.5X10^4^	40	0	Positive	0.1	-	31	619
4	8	-	4	-	5	160	0	40	0	0	0.1	0.1	54	-
5	4	-	4	-	5	160	0	40	0	0	0.1	0.1	60	-
6	16	-	4	-	7.5	-	0	40	0	0	0.2	0.1	51	-
7	16	-	16	-	28.2	-	0	80	0	0	0.1	0.1	60	-

**Figure 1:**
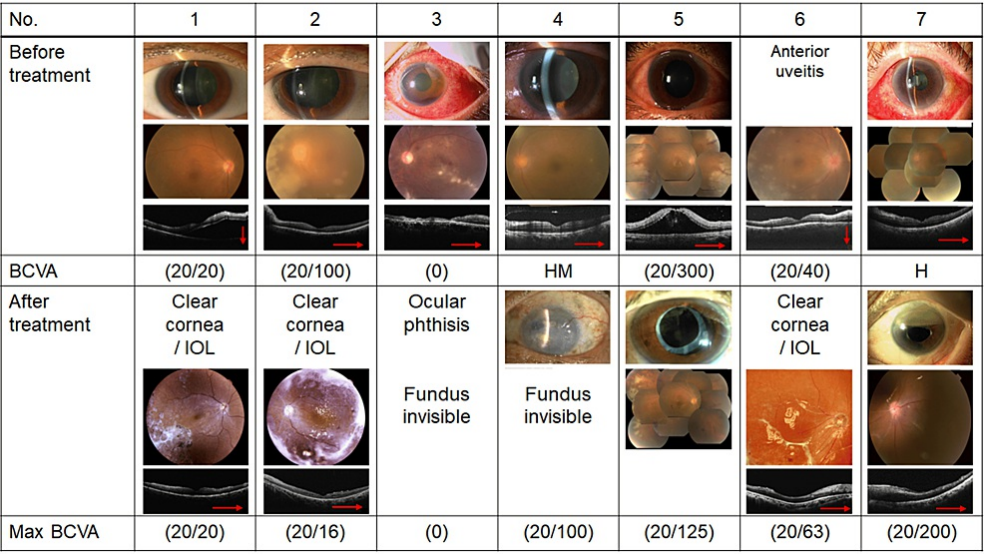
The anterior eye, retinal findings, and best-corrected visual acuity (BCVA) before and after treatment BCVA: best-corrected visual acuity; IOL: intraocular lens

Figure 1 shows the findings of the anterior eye and retina before and after treatment. Table 3 shows the clinical features of patients with ARN. Intraocular lesions during the course of the disease included ocular hypertension in one eye (14.3%), granulomatous anterior uveitis in seven eyes (100.0%), retinal hemorrhage in five eyes (71.4%), vitreous opacity in seven eyes (100.0%), peripheral retinal vasculopathy with yellowish-white retinal exudative lesions in six eyes (85.7%), optic nerve atrophy in six eyes (85.7%), retinal vascular occlusion in six eyes (85.7%), retinal choroidal degeneration and atrophy in six eyes (85.7%), subretinal macular fluid in five eyes (71.4%), and choroidal detachment in six eyes (85.7%). Macular edema was observed in seven eyes (100.0%).

**Table 3:** Presence of ARN-specific ocular finding ARN: acute retinal necrosis; SRD: serous retinal detachment; PED: retinal pigment epithelial detachment; CME: cystoid macular edema

Eye No.		Acute phase of ARN							Late phase of ARN							
	Anterior uveitis		Ocular hypertension	Retinal hemorrhage	Vitreous opacity	Retinal exudative vasculopathy		Retinal detachment		Optic nerve atrophy	Retinal vascular occlusion	Chorioretinal Degeneration /atrophy	SRD	PED	CME	
1	1		0	0	1	1		1		0	0	0	0	0	1	
2	1		0	1	1	1		0		1	1	1	1	0	1	
3	1		0	1	1	1		1		1	1	1	1	0	1	
4	1		0	1	1	1		1		1	1	1	0	0	1	
5	1		0	1	1	1		1		1	1	1	1	0	1	
6	1		1	1	1	1		1		1	1	1	1	0	1	
7	1		0	0	1	0		1		1	1	1	1	0	1	
Total	7		1	5	7	6		6		6	6	6	5	0	7	
(%)	(100.0)		(14.3)	(71.4)	(100.0)	(85.7)		(85.7)		(85.7)	(85.7)	(85.7)	(71.4)	(0.0)	(100.0)	

The severity classifications are presented in Table 4. Six of the seven eyes were classified as fulminant and one as severe.

**Table 4:** Severity classification of ARN* * Modified classification of ARN severity by Tokyo Medical University [13] ARN: acute retinal necrosis

Classification	Findings	The number of eye
Mild type	Localized retinal lesions with slow progression	0
Severe type	Entire retinal lesions with slow progression	1
Fulminant type	Yellowish-white retinal lesions extending to the posterior pole within 10 days of onset	6

Details of the medical therapy are shown in Table 5. The mean time from symptom onset to the initiation of medical therapy was 10.3 ± 9.2 days. Treatment consisted of oral and intravenous antiviral drugs in six eyes (85.7%), oral antiplatelet drugs in six eyes (85.7%), steroid eye drops in six eyes (85.7%), steroid injection (subcapsular tenon) in four eyes (57.1%), vitreous in three eyes (42.9%), oral steroids in five eyes (71.4%), and surgical treatment in six eyes (85.7%). Oral prednisolone was used for approximately three months, starting at a dose of 30-40 mg per day and gradually decreasing in five eyes. Oral antivirals were administered for seven days. Medical therapy included 0.1% betamethasone sodium phosphate solution in six of the seven eyes, two types of antiglaucoma eye drops in one of the seven eyes, and prednisolone in five of the seven eyes. Oral prednisolone was administered to five of the seven eyes. Subtenon injections of triamcinolone acetonide (TA) were used in two eyes and dexamethasone sodium phosphate in two eyes. Oral antivirals were given for seven days. Oral antivirals were 1000 mg/day for acyclovir (ACV: Zovirax®) in two eyes, 500-1000 mg/day for valacyclovir hydrochloride (VACV: Valtrex®) in two eyes, and 750 mg/day for famciclovir (FCV: Famvir®) in two eyes. Subtenon injections of steroids and oral antivirals were treated simultaneously. Two eyes received intravitreal injections of TA in Eye No. 6 and 7. Aspirin (Biaspirin tablet®) was administered to six of seven eyes. Intravenous infusions of ACV (30 mg/kg/day) were administered to five of the seven eyes for two to three weeks. Eye No. 3 tested positive for CMV and HIV and was treated with valganciclovir hydrochloride (Varixa®) and a sulfamethoxazole/trimethoprim combination (Bacta®). Eye No. 3 did not receive intravitreal antiviral therapy because treatment for CMV and HIV was prioritized.

**Table 5:** Medical therapy PSL: prednisolone; BM: 0.1%: betamethasone sodium phosphate solution; DEX: dexamethasone sodium phosphate; TA: triamcinolone acetonide; FCV: famciclovir; ACV: acyclovir; VACV: valacyclovir hydrochloride

Eye No.	Time interval from the first symptom to treatment (days)	Corticosteroid					Antivirals		Antiplatelet
		Eye drop	Subcapsular tenon injuection	Intrevitreous injection	Oral PSL (per day)	Intravenous	Oral	Intravenous	Oral aspirin
1	12	BM	DEX	―	30 mg	PSL	FCV	ACV	Aspirin
2	12	BM	DEX	TA	30 mg	PSL	FCV	ACV	Aspirin
3	3	BM	―	―	―	―	ACV	―	Aspirin
4	10	BM	―	TA	40 mg	PSL	VACV	ACV	Aspirin
5	4	BM	―	―	30 mg	PSL	VACV	ACV	Aspirin
6	1	BM	TA	―	―	―	ACV	ACV	Aspirin
7	30	BM	TA	TA	30㎎	―	―	―	―

Table 6 presents the details of the ocular surgeries. Pars plana vitrectomy (PPV) was performed in five eyes and cataract surgery alone in one eye. The mean time from PPV to surgery was 38 ± 32 days. Of the five eyes that underwent PPV, five eyes (80%) underwent silicone oil replacement, two (40%) underwent ring closure, and four (80%) underwent intraocular lens implantation. Retinal reattachment was achieved in all six eyes that underwent surgery. The postoperative best-corrected visual acuity (BCVA) was 20/200 or better in four eyes.

**Table 6:** Surgical details BCVA: best-corrected visual acuity; HM: hand motion; IOL: intraocular lens

Eye No.	Preoperative		Surgical procedure					Postoperative	
	BCVA	Time from onset of ARN to surgery (days)	Pars plana vitrectomy	Encircling scleral buckle	Tamponade (silicone oil)	Cataract surgery	IOL implant	BCVA	Retina complete flat
1	(20/50)	97	1	0	1	1	1	(20/20)	1
2	(20/63)	20	1	0	1	1	1	(20/16)	1
3	No surgery								
4	(20/250)	22	1	0	1	1	1	(20/100)	1
5	(20/2000)	787	0	0	0	1	1	(20/124)	1
6	(20/600)	6	1	1	1	1	1	(20/63)	1
7	(HM)	45	1	1	1	1	0	(20/2000)	1
Total	―	―	5 eyes	2 eyes	5 eyes	6 eyes	5 eyes	―	6 eyes

The results of polymerase chain reaction (PCR) detection of intraocular viral deoxyribonucleic acid (DNA) are shown in Table 7. Anterior chamber fluid was collected from one eye, and vitreous fluid was collected from four eyes. CMV DNA was detected in the anterior chamber fluid of one eye, and VZV DNA was detected in the vitreous fluid of three eyes.

**Table 7:** Polymerase chain reaction (PCR) of viral DNA PCR: polymerase chain reaction; DNA: deoxyribonucleic acid, IL2-R: interleukin-2 receptor; HSV: herpes simplex virus; VZV: varicella-zoster virus; CMV: cytomegalovirus; -: not tested

Eye No.	Vitrectomy	Aquaous humor fluid (virus PCR)			Vitreous samples（virus PCR and IL-2R)		
		HSV-DNA	VZV-DNA	CMV-DNA	HSV-DNA	VZV-DNA	IL-2R (U/ml)
1	＋	-	-	-	Negative	Positive	-
2	＋	-	-	-	Negative	Positive	-
3	-	Negative	Negative	Positive	-	-	-
4	＋	-	-	-	-	-	-
5	-	-	-	-	-	-	-
6	＋	-	-	-	Negative	-	-
7	＋	-	-	-	Negative	Positive	266

The refractive values at baseline and progression of BCVA are shown in Table 8. Three eyes had a BCVA of less than 20/200 at baseline, and four eyes had a BCVA of less than 20/200 at the last visit. At the last visit, the mean Logarithm of the Minimum Angle of Resolution (LogMAR) BCVA was 1.29, which tended to decrease compared with 0.77 BCVA at the first visit, but there was no significant difference between the two.

**Table 8:** Refractive values at baseline and progression of BCVA BCVA: best-corrected visual acuity; HM: hand motion; CF: counting fingers

Eye No.	Severity classification	Before treatment				After treatment	
		Refractive error (diopters)	BCVA at baseline	BCVA before treatment	Max BCVA after treatment		Final BCVA
1	Fulminant type	-3.88	(20/16)	(20/20)	(20/20)		(20/20)
2	Fulminant type	-3.75	(20/63)	(20/100)	(20/16)		(20/100)
3	Fulminant type	-6.88	(20/2000)	0	0		0
4	Fulminant type	0.75	(20/16)	HM	(20/100)		HM
5	Fulminant type	-0.50	(20/300)	(20/300)	(20/125)		(20/125)
6	Fulminant type	-0.50	(20/40)	(20/40)	(20/63)		(20/600)
7	Severe type	-1.75	(20/600)	HM	(20/2000)		CF

Table 9 shows a comparison of the results of this study with reports of studies of ARN from other centers in Japan [5,6,7,8].

**Table 9:** Comparison with other reports of ARN in Japan BCVA: best-corrected visual acuity

Name of University	Hirosaki Univ. [5]	Tokyo Medical Univ. [6]	Hokkaido Univ. [7]	Kyorin Univ. [8]	Teikyo Univ. (This study)
Years	2003	2007	2008	2011	2024
Number of patients and eyes	12 patients (14 eyes)	80 patients (84 eyes)	19 patients (21 eyes)	14 patients (16 eyes)	6 patients (7 eyes)
Classification severe and fulminant	72%	70%	53%	71%	100%
Retinal detachment	72%	61%	38%	43％	85.7%
Final BCVA less than (20/200)	43%	43%	43%	21%	57%

## Discussion

Summary of the study

Six of the seven eyes were fulminant, and one eye was severely affected, with more cases being extremely severe compared with those in other reports. The mean time from symptom onset to treatment initiation was 10.3 days, and treatment was initiated relatively early; however, the final BCVA was worse (20/200) in four of the seven eyes. Retinal inflammation resolved in five eyes that underwent PPV and one eye that received medical therapy only. In one eye, surgery was not indicated because it was considered difficult to restore visual acuity. Patients with poor pre-treatment visual acuity had poor final visual acuity, emphasizing the importance of early detection and treatment, including PPV.

Patient background factors

The mean age of the patients at the time of their first visit was 63.6 years. Except for the patient (Eye No. 3), who was 43 years old, most of the patients were relatively old. Regarding systemic diseases, three of the six patients had hypertension, and one of the six had cerebral ischemic disease. However, none of the patients were immunocompromised. Eye No. 3 was also a healthy patient, with only retinitis. In Eye No. 3, serologic testing for the diagnosis of retinitis turned out to be positive for HIV. This was added to the results section. In a previous meta-analysis of retrospective cohort studies on the background of ARN cases, the proportion of immunocompromised patients was as low as 21% in 24 patients with ARN, whereas it was as high as 100% in 14 cases of CMV retinitis and three cases of progressive outer retinal necrosis (PORN) [14]. Our patients were mostly healthy, except for one case (Eye No. 3). When retinitis develops in immunocompromised patients, viral retinitis is suspected, allowing the early administration of antiviral drugs. However, in the case of retinitis in healthy participants, there is a concern that treatment initiation may be delayed because of the time required for testing. Therefore, it has been proposed to clinically suspect ARN and intervene in the treatment of patients with retinitis without waiting for the PCR detection of viral DNA [14].

A large Japanese multicenter study of the clinical manifestations in 149 cases of ARN found that the findings at initial presentation were anterior chamber cells or mutton-fat keratic precipitates (97%) and unilateral (93%) or yellow-white retinal lesions (86%) [15]. The clinical course of ARN was characterized by a rapid overall expansion of retinal lesions (39%), the development of retinal break or RD (55%), and optic nerve atrophy (43%). Among these, four variables were identified as being associated with poor visual prognosis in ARN: posterior iris synechia, initial visual acuity, VZV infection, and retinal phlebitis [15]. Therefore, if ARN is suspected based on clinical findings and these parameters are associated with poor visual prognosis, it may be effective to initiate aggressive antiviral therapy before the results of PCR detection of intravital viral DNA in the anterior chamber are available [15].

Retinal findings characteristic of ARN

In our case series, the retinal findings were considered characteristic of ARN (fundus hemorrhage, vitreous opacity, multifocal exudative yellow-white peripheral retinitis, optic nerve atrophy, retinal vascular occlusion, retinal choroidal degeneration/atrophy, serous RD, and cystoid macular edema). ARN is necrotizing retinitis that affects the periphery of the retina. Necrotizing retinitis is characterized by full-thickness retinal necrosis with or without inflammation [12]. Retinal healing leaves atrophic, collagenous, and gliotic scars in the affected retinal areas [12]. Clinically, the initial presentation is white to yellow retinal edema and opacity, with or without hemorrhage [12]. The diagnostic ARN criteria by the Standardization of Uveitis Nomenclature (SUN) Working Group mandate the presence of peripheral necrotizing retinitis, coupled with either conclusive verification of intraocular infection attributed to HSV or VZV through PCR analysis of intraocular fluid or manifestation of classical clinical features characteristic of ARN [12].

In 2015, the Japanese ARN Study Group proposed ARN criteria with two levels of certainty: virus-confirmed and virus-unconfirmed. Both virus-confirmed and virus-unconfirmed conditions require anterior segment inflammation, yellowish-white periretinal retinal lesions, and continued observation to determine progression [16]. If the virus is confirmed, one of the following symptoms is required to diagnose ARN: rapid total retinal expansion, development of a retinal break or RD, retinal vascular occlusion, development of optic nerve atrophy, or response to antiviral therapy. If the virus is unconfirmed, any two of the above and any two additional clinical manifestations, including retinal arteritis, optic disc hyperemia, vitritis, and elevated intraocular pressure, are required [16]. In our case, we observed a high rate of fundus hemorrhage, vitreous opacity, and perifoveal retinal exudative vasculopathy in the acute phase, as well as optic nerve atrophy, retinal vascular occlusion, and retinal choroidal degeneration/atrophy in the late treatment phase, which have high diagnostic value for the clinical diagnosis of ARN.

Medical therapy

Treatments for ARN include medical, laser, and surgical therapies. The initial antiviral therapy includes intravenous administration [17]. Our patient's medical therapy included the basic administration of 0.1% betamethasone sodium phosphate as eye drops and subtenon and vitreous injections of TA. Prednisolone, antivirals (ACV, VACV, and FCV), and anti-platelet agents (aspirin) were used orally. Intravenous infusions were predominantly mixtures of ACV and prednisone. ACV, or oral VACV, and other systemic agents such as FCV and valganciclovir, as well as intravenous foscarnet or ganciclovir, are recommended worldwide as standard treatment for ARN [18].

In our case, standardized antiviral and anti-inflammatory treatments for ARN were administered, but most patients experienced vitreous opacity and intraocular inflammation; only one eye resolved with medication, but in six eyes, ARN activity was not suppressed and PPV was required in five eyes. In cases of VZV or CMV retinitis, acyclovir does not yield significant improvement; thus, ganciclovir or valganciclovir is recommended instead [19]. It is also generally reported that patients with ARN caused by VZV tend to be older, whereas those with ARN caused by HSV tend to be younger [20,21]. Our third patient (Eye No. 3) was younger (43 years old) and had more significant VZV and HIV than HSV in his serum. Therefore, valganciclovir hydrochloride and a sulfamethoxazole/trimethoprim combination were administered. However, the retinitis did not resolve. Finally, CMV was detected in the patient's anterior chamber fluid. This reaffirms the need for early administration of ganciclovir or valganciclovir in young patients with significant serum VZV.

In addition to antiviral therapy, early treatment with oral corticosteroids after antiviral therapy is recommended to minimize the risk of retinal inflammation and rhegmatogenous RD (RRD) [17].

Retrospective clinical studies from Korea have shown that laser photocoagulation is also effective in preventing RRD in ARN [22]. However, a meta-analysis of retrospective cohort studies concluded that there is no convincing evidence for the efficacy of prophylactic lasers in preventing RRD [23]. A cohort study by the same group also reported that prophylactic PPV significantly reduced the risk of RRD due to ARN compared to antiviral treatment, but long-term silicone oil tamponade caused significant visual loss [24]. However, whether early PPV should be performed to prevent RRD remains inconclusive [24,25].

Viral antibody titers and serologic tests

Viral antibody titers and serologic tests at the beginning of treatment showed positive HSV1, VZV, and CMV antibody titers in all patients; however, the titers were not very high. Of the five vitrectomized eyes, PCR testing was performed in four eyes; VZV DNA was detected in three of the four eyes. Currently, PCR detection of viral DNA in aqueous humor fluid or vitreous samples and PPV biopsy are effective methods for diagnosing ARN [26]. Early diagnosis and identification of the type of viral infection can lead to the early initiation of antiviral therapy for ARN and improve retinitis and visual prognosis [27].

Intraocular viral PCR should have been performed in all cases; however, in our case series, testing was not performed in one of the five eyes that underwent PPV. In Eye No. 3, PPV was not performed; however, PCR was performed to detect the virus in the anterior chamber fluid. The results were positive for CMV DNA. Ultimately, four of the five eyes tested positive for viral DNA, including three eyes with VZV DNA and one eye with CMV. The serum HIV was also positive in the patient (Eye No. 3), suggesting that this case is a partial overlap of ARN, CMV retinitis, and PORN.

Prior investigations concerning the detection of viral DNA in ARN through PCR analysis warrant careful consideration. A study conducted in France that examined aqueous humor fluid or vitreous samples from 30 ARN-afflicted eyes revealed compelling findings: VZV DNA was detected in 15 eyes (50%, median age 57 years), HSV-1 DNA in seven eyes (23%, median age 47 years), HSV-2 DNA in six eyes (20%, median age 20 years), and CMV DNA in one eye (3%) [28]. Moreover, a comprehensive meta-analysis of retrospective cohort studies revealed pertinent insights: among 26 eyes afflicted with ARN, VZV infection was identified in 46% (n=12), HSV-1 in 23% (n=6), and HSV-2 in 31% (n=8) [14]. Consequently, within our present case series, a noteworthy prevalence of VZV infection superseded that of HSV infection in ARN, thus aligning with established epidemiological trends [14,21,28].

Surgical procedures

PPV was performed in five of the seven eyes, whereas cataract surgery alone was performed in one eye. Among the five eyes subjected to PPV, all underwent silicone oil tamponade (100%), and two eyes (40%) additionally underwent encircling scleral buckling. Consequently, complete postoperative retinal restoration was achieved in all surgically treated eyes. The objectives and associated benefits of performing PPV for ARN include: removing the vitreous scaffold to remove inflammatory mediators and viruses; removing vitreous traction and preventing RD; using silicone oil tamponade after PPV to prevent further tractional RD; and administering laser, antiviral, and steroid medications as needed during surgery.

While extensive literature exists detailing post-PPV outcomes in ARN management, the majority comprise retrospective studies owing to the exigency and assertiveness requisite in treating ARN, rendering interventional studies challenging. Additionally, postoperative prognoses are subject to variation contingent upon the timing of PPV initiation, the surgical technique employed, and the pre-treatment severity of ARN [17]. In one of the few interventional inquiries, a comparison was conducted between 20 eyes treated solely with intravitreal acyclovir and oral prednisolone and 10 eyes treated with early PPV accompanied by intravitreal acyclovir lavage [25]. Notably, a higher incidence of RD was observed in the medical treatment cohort, with 18 of 20 eyes affected by ARN, as opposed to the early PPV group, in which RD occurred in four of 10 eyes. Nevertheless, no statistically significant difference was observed in the mean final BCVA between the two groups [25]. In contrast, a study from China reported that 16 eyes in the early prophylactic PPV ARN group had both reduced RD and improved final visual acuity compared with 21 eyes with ARN receiving the usual treatment (medical treatment followed by PPV as needed) [29]. Similarly, a comparative study in Texas, USA, reported that early PPV for ARN within 30 days prevented RD after ARN [30]. However, other studies from the Asian region have reported that prophylactic PPV shows no difference in RD recurrence rates or BCVA improvement, and consequently, PPV does not improve the prognosis of ARN [31, 32]. On the other hand, a recent retrospective clinical study of 63 eyes in the Netherlands showed that the risk of RRD was highest in 33 patients who received prophylactic laser (45.5%), lower in 15 patients who did not receive prophylaxis (26.7%), and lowest in seven patients who received prophylactic PPV (14.3%) and the seven patients who underwent prophylactic PPV [33]. Taken together, the results of these clinical studies conclude that prophylactic laser can be abandoned for ARN; however, whether prophylactic PPV leads to the prevention of RD or improved visual prognosis still needs to be investigated [33].

Despite the implementation of appropriate therapeutic modalities, RD remains prevalent in patients with ARN. The standard procedural approach for PPV typically entails meticulous peripheral PPV coupled with endolaser application targeting necrotic retinal regions and intravitreal administration of antiviral agents. Furthermore, silicone oil tamponade is favored over long-acting gas replacements as it is deemed more efficacious in concluding surgical procedures. Notably, silicone oil tamponade serves the dual purpose of preventing the onset of proliferative vitreoretinopathy, a complication known to manifest at a high frequency after PPV surgery for ARN. A seminal case series encompassing 32 ARN-afflicted eyes revealed that 15 eyes developed RD, of which 13 underwent vitrectomy; notably, six of these eyes (46%) had recurrent RD [34]. In light of these findings, we advocate the inclusion of silicone oil tamponade during the initial PPV following comprehensive vitreous dissection, with adjunct consideration of scleral buckle grafting if deemed necessary.

Progress of visual acuity

Comparing our report with that of other universities in Japan, the BCVA of less than (20/200) was 21-43% in other universities, while it was much higher in our case at 57% [5,6,7,8]. This is because the total number of fulminant and severe forms in our case series was high (100%), while the number of cases was small (seven eyes).

In a study comparing final LogMAR BCVA after treatment of ARN, PORN, and CMV retinitis, patients with PORN (1.65 ± 1.34 LogMAR) tended to have lower BCVA than those with ARN (0.89 ± 0.73 LogMAR) and CMV (0.65 ± 0.59 LogMAR), but no differences between the three groups were reported [14]. In patients with ARN, the pre-treatment BCVA was less than (20/200 = 1 LogMAR) in seven of 11 eyes, and the final BCVA was also less than (20/200 = 1 LogMAR) in eight of 11 eyes, suggesting that overall visual recovery is difficult [35]. On the other hand, a report of 13 eyes that underwent 25-gauge PPV for RD associated with ARN showed improvement from preoperative BCVA (2.03 ± 0.29 LogMAR) to postoperative BCVA (1.57 ± 0.63 LogMAR), six eyes (42.9%) had restored retinal function, and nine eyes (64.3%) had improved visual acuity [36]. The authors reported that pre-treatment visual acuity was better than 1 LogMAR in zero of 13 eyes (0%), and the final visual acuity was in five of 13 eyes [36]. Final visual acuity results vary among reports because different parameters, such as disease severity, pre-treatment visual acuity, time from disease onset to treatment, and treatment modality, affect treatment effectiveness. Therefore, a large-scale multicenter registry study is required to establish the guidelines for ARN treatment.

Limitation

In this study, the patient selection process relied on individuals whose medical records explicitly documented an ARN diagnosis. Consequently, there is the potential for exclusion of patients who received treatment for ARN but were not explicitly labeled as such in their medical records.

## Conclusions

This article focuses on ARN cases and reviews their clinical presentation, treatment course, and prognosis. ARN is a serious ocular disease that causes visual impairment, so understanding and appropriate treatment are critical. The final visual acuity tended to be worse than the initial visual acuity, but the difference was not statistically significant. However, patients with poor visual acuity before treatment tended to have worse final visual acuity. These findings emphasize the importance of early diagnosis and treatment. However, the prognosis of ARN treatment remains poor. The accumulation of cases from many institutions, as in our case, may lead to improved treatment strategies.
